# Supplementary material for: Germline status and micronutrient availability regulate a somatic mitochondrial quality control pathway via short-chain fatty acid metabolism
Source: bioRxiv. 2024 May 21:2024.05.20.594820. Preprint. [Version 1] doi: 10.1101/2024.05.20.594820 (PMC11142046; doi:10.1101/2024.05.20.594820)
Supplement: Supplement 2 [file NIHPP2024.05.20.594820v1-supplement-2.pdf]

bioRxiv preprint doi: https://doi.org/10.1101/2024.05.20.594820; this version posted May 21, 2024. The copyright holder for this preprint (which was not certified by peer review) is the author/funder, who has granted bioRxiv a license to display the preprint in perpetuity. It is made available under aCC-BY 4.0 International license.

SUPPLEMENTAL ONLY REFERENCES

S1. Kamath, R.S., Fraser, A.G., Dong, Y., Poulin, G., Durbin, R., Gotta, M., Kanapin, A., Le Bot, N., Moreno, S., Sohrmann, M., et al. (2003). Systematic functional analysis of the Caenorhabditis elegans genome using RNAi. Nature 421, 231–237. 10.1038/nature01278.

S2. Dokshin, G.A., Ghanta, K.S., Piscopo, K.M., and Mello, C.C. (2018). Robust Genome Editing with Short Single-Stranded and Long, Partially Single-Stranded DNA Donors in Caenorhabditis elegans. Genetics 210, 781–787. 10.1534/genetics.118.301532.

bioRxiv preprint doi: https://doi.org/10.1101/2024.05.20.594820; this version posted May 21, 2024. The copyright holder for this preprint (which was not certified by peer review) is the author/funder, who has granted bioRxiv a license to display the preprint in perpetuity. It is made available under aCC-BY 4.0 International license.

S3. Paix, A., Folkmann, A., Rasoloson, D., and Seydoux, G. (2015). High Efficiency, Homology-Directed Genome Editing in Caenorhabditis elegans Using CRISPR-Cas9 Ribonucleoprotein Complexes. Genetics 201, 47–54. 10.1534/genetics.115.179382.

S4. Bolger, A.M., Lohse, M., and Usadel, B. (2014). Trimmomatic: a flexible trimmer for Illumina sequence data. Bioinformatics 30, 2114–2120. 10.1093/bioinformatics/btu170.

S5. Dobin, A., Davis, C.A., Schlesinger, F., Drenkow, J., Zaleski, C., Jha, S., Batut, P., Chaisson, M., and Gingeras, T.R. (2013). STAR: ultrafast universal RNA-seq aligner. Bioinformatics 29, 15–21. 10.1093/bioinformatics/bts635.

S6. Patro, R., Duggal, G., Love, M.I., Irizarry, R.A., and Kingsford, C. (2017). Salmon provides fast and bias-aware quantification of transcript expression. Nat. Methods 14, 417–419. 10.1038/nmeth.4197.

S7. Soneson, C., Love, M.I., and Robinson, M.D. (2016). Differential analyses for RNA-seq: transcript-level estimates improve gene-level inferences. Preprint at F1000Research, 10.12688/f1000research.7563.2 10.12688/f1000research.7563.2.

S8. Love, M.I., Huber, W., and Anders, S. (2014). Moderated estimation of fold change and dispersion for RNA-seq data with DESeq2. Genome Biol. 15, 1–21. 10.1186/s13059-014-0550-8.

S9. Szklarczyk, D., Kirsch, R., Koutrouli, M., Nastou, K., Mehryary, F., Hachilif, R., Gable, A.L., Fang, T., Doncheva, N.T., Pyysalo, S., et al. (2023). The STRING database in 2023: protein–protein association networks and functional enrichment analyses for any sequenced genome of interest. Nucleic Acids Res. 51, D638–D646. 10.1093/nar/gkac1000.

S10. Brenner, S. (1974). THE GENETICS OF CAENORHABDITIS ELEGANS. Genetics 77, 71–94. 10.1093/genetics/77.1.71.

S11. Shoura, M.J., Gabdank, I., Hansen, L., Merker, J., Gotlib, J., Levene, S.D., and Fire, A.Z. (2017). Intricate and Cell Type-Specific Populations of Endogenous Circular DNA (eccDNA) in Caenorhabditis elegans and Homo sapiens. G3 GenesGenomesGenetics 7, 3295–3303. 10.1534/g3.117.300141.

S12. Grub, L.K., Held, J.P., Hansen, T.J., Schaffner, S.H., Canter, M.R., Malagise, E.M., and Patel, M.R. (2023). A role for N6-methyldeoxyadenosine in C. elegans mitochondrial genome regulation. Preprint at bioRxiv, 10.1101/2023.03.27.534452 10.1101/2023.03.27.534452.

S13. Gitschlag, B.L., Kirby, C.S., Samuels, D.C., Gangula, R.D., Mallal, S.A., and Patel, M.R. (2016). Homeostatic Responses Regulate Selfish Mitochondrial Genome Dynamics in C. elegans. Cell Metab. 24, 91–103. 10.1016/j.cmet.2016.06.008.
